# Population genomic analyses reveal hybridization and marked differences in genetic structure of *Scurria* limpet sister species with parapatric distributions across the South Eastern Pacific

**DOI:** 10.1002/ece3.8888

**Published:** 2022-05-07

**Authors:** Pablo Saenz‐Agudelo, Lívia Peluso, Roberto Nespolo, Bernardo R. Broitman, Pilar A. Haye, Marco A. Lardies

**Affiliations:** ^1^ 28033 Instituto de Ciencias Ambientales y Evolutivas Universidad Austral de Chile Valdivia Chile; ^2^ ANID‐ Millennium Science Initiative Nucleus (NUTME) Las Cruces Chile; ^3^ 28033 Doctorado en Ciencias Mención Ecología y Evolución Escuela de Graduados Facultad de Ciencias Universidad Austral de Chile Valdivia Chile; ^4^ ANID‐ Millennium Science Initiative Nucleus (LiLi) Valdivia Chile; ^5^ Center for Applied Ecology and Sustainability (CAPES) Santiago Chile; ^6^ Millennium Institute for Integrative Biology (iBio) Santiago Chile; ^7^ Departamento de Ciencias Facultad de Artes Liberales Universidad Adolfo Ibañez Santiago Chile; ^8^ ANID‐ Millennium Science Initiative Nucleus UPWELL Santiago Chile; ^9^ Instituto Milenio en Socio‐Ecología Costera (SECOS) Santiago Chile; ^10^ Departamento de Biología Marina Universidad Católica del Norte Coquimbo Chile

**Keywords:** intertidal, parapatry, population genomics, RADseq, *Scurria*, South Eastern Pacific

## Abstract

The study of sister species that occur in parapatry around biogeographic transition zones can help understand the evolutionary processes that underlie the changes in species composition across biogeographic transition zones. The South Eastern Pacific (SEP) coast is a highly productive coastal system that exhibits a broad biogeographic transition zone around 30–35°S. Here, we present a comparative genome‐wide analysis of the sister species *Scurria viridula* and *Scurria zebrina*, that occur in parapatry and whose poleward and equatorward range edges intersect in the 30–35°S SEP biogeographic transition zone. We sampled 118 specimens sourced from nine sites from Tocopilla (22°S) to Chiloé (41°S) including one site where both species overlap and analyzed over 8000 biallelic single nucleotide polymorphisms. We found evidence of hybridization between these species in the contact zone and found significant but contrasting population structures for both species. Our results indicate that the genetic structure in *S*. *viridula*, which is currently expanding its range poleward, follows a simple isolation by distance model with no traces of natural selection (no evidence of outlier loci). In contrast, *S*. *zebrina*, which has  its equatorward range edge at the transition zone, displayed a pronounced genetic break approximately at 32–34°S, along a region of marked environmental heterogeneity in association with a semi‐permanent coastal upwelling regime. For *S*. *zebrina* we also found 43 outlier loci associated with this genetic break, with a significant proportion of them clustering in a single linkage group. This marked difference in the presence of outlier loci between species suggests that they could be responding differently to local environmental challenges found at their overlapping geographic range edges, thus providing important new insights about genomic changes around biogeographic transition zones in sister species and the forces that shape genetic diversity in intertidal marine species.

## Graphical abstract

Here, we report a novel comparative population genetics study of two parapatric sister marine limpets endemic to the South Eastern Pacific and whose poleward and equatorward range edges intersect in the 30–35°S SEP biogeographic transition zone. We used RADseq to survey thousands of SNPs along with the distribution of both species, including one locality where they overlap. Our results provide evidence of hybridization between species and contrasting patterns of genetic structure between species. These species appear to be responding differently to local environmental challenges found at their overlapping geographic range edges, thus providing important new insights about genomic changes around biogeographic transition zones in sister species and the forces that shape genetic diversity in intertidal marine species.

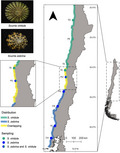

## 1 INTRODUCTION

The world's oceans are in stark contrast to most terrestrial ecosystems as they are embedded in a water matrix with very few physical barriers that can prevent dispersal and where most organisms have at least one mobile phase during their life cycle that is suited for long‐distance dispersal (Hellberg, 2009; Kinlan et al., 2005; Lester et al., 2007; Shaw et al., 2019). Despite this high potential for dispersal, species distribution is far from being homogeneous, which is attested by the rich number of biogeographic transitions described to date (Spalding et al., 2007). Transition zones between biogeographic regions are paradigmatic, representing good systems for studying the origin of marine biodiversity (Angert et al., 2020; Dawson et al., 2002; Haye et al., 2014; Johannesson et al., 2020; Kerr & Alroy, 2021). These zones often display important changes in species composition, and for species that are present across them, changes in species abundance, or changes in the genetic composition (genetic structure) are also commonly reported (Bowen et al., 2016; Golla et al., 2020). In this sense, comparative studies of sister species that are in contact over a transition zone offer a good opportunity to explore the historic and evolutionary processes associated with their population divergence (Johannesson et al., 2020).

The South Eastern Pacific (SEP) extends over 6000 km from northern Peru to the southern tip of South America. It is a heterogeneous seascape characterized by an intense and spatially structured coastal upwelling circulation (Aravena et al., 2014) that makes it one of the most productive marine ecosystems on the planet (Thiel et al., 2007). This region is also characterized by a complex biodiversity distribution that inspired several authors to define different biogeographic units (see Lara et al., 2019). Possibly the most widely accepted division is the one by Camus, 2001, with two biogeographic provinces with distinct species assemblages separated by a transition zone. The Peruvian Province extends from 4 to 30°S, the transition zone called the intermediate area expands from 30 to 42°S and the Magellanic Province expands from 42°S down to 56°S (Camus, 2001). Much attention has been given to the biogeographic break located at 30°S that separates the Peruvian province from the intermediate area. This break is the distributional boundary for many species (both equatorward and poleward) and has been documented as an effective barrier to gene flow for some species that have distributions that cross this break (for examples see Haye et al., 2014, 2019; Montecinos et al., 2012; Tellier et al., 2009) but not for others (such as in Cárdenas et al., 2009; Rojas‐Hernandez et al., 2016). In addition, long‐term surveys have shown that environmental characteristics, particularly upwelling, not only are highly heterogeneous along the Chilean Pacific coast but also change dramatically across the 30°S latitude (Aravena et al., 2014; Broitman et al., 2018; Lara et al., 2019; Torres et al., 2011). Thus, populations of wild benthic organisms inhabiting this region can experience differences in their exposure to coastal upwelling. These observations have fueled different hypotheses regarding the role of this break as a barrier to gene flow and expansion of species distribution. Yet the mechanisms and underlying evolutionary processes behind these patterns remain unclear. It is in this geographic context that the limpets of the genus *Scurria* are found.

**FIGURE 1:**
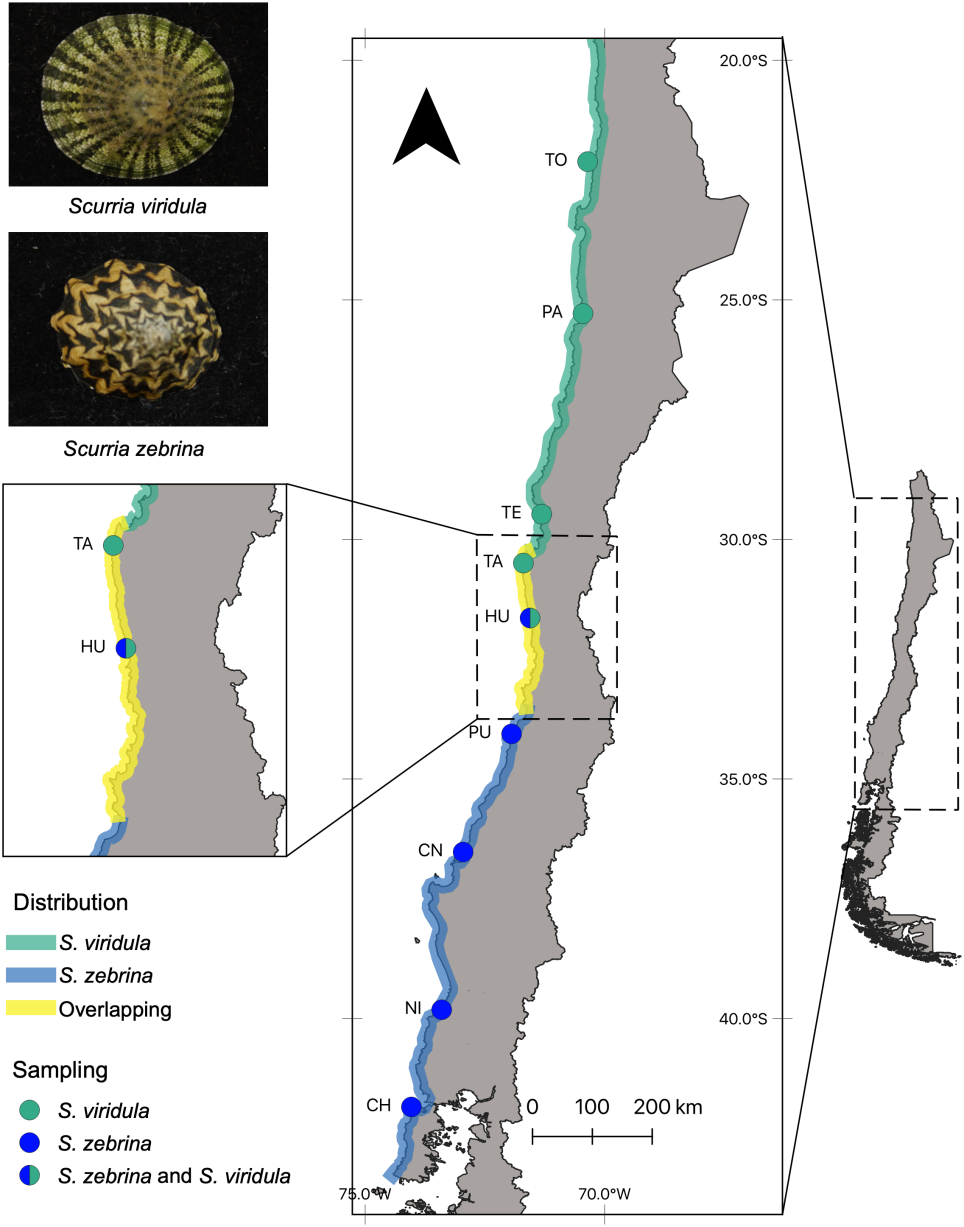
Map showing the location of the distribution and sampling sites for this study

The genus *Scurria* (Gray, 1847) belongs to the most ancestral group of living Gastropods, the order Patellogastropoda (Nakano & Ozawa, 2007; Nakano & Sasaki, 2011). Patellogastropod limpets are abundant inhabitants of intertidal and subtidal rocky shores throughout the global oceans and play important ecological roles in littoral marine ecosystems (Aguilera et al., 2013; Espoz et al., 2004; Nakano & Ozawa, 2007). The genus *Scurria* encompasses 8 described species based on morphology and DNA (16S) data, all endemic to the SEP (Espoz et al., 2004), but a recent unpublished work using six molecular markers suggests the presence of 10 species in this group (Asorey, 2017). Here, we present the results of a comparative population genetic survey of *Scurria viridula* (Lamark, 1819) and *Scurria zebrina* (Lesson, 1830). These sister species diverged approximately 15 My ago (Nakano & Ozawa, 2007) and occur in parapatry along the SEP coast of Chile. *S viridula* is distributed along the Peruvian province between 12 and 33°S, while *S*. *zebrina* is distributed along the transition zone between 31 and 42°S (Figure 1). Both species overlap on a narrow zone of approximately 250 km around the 31–33°S transition zone (Aguilera et al., 2020). Previous studies indicate that this overlap is recent and due to the southwards range expansion of *S*. *viridula* in the last 50 years (Rivadeneira & Fernández, 2005). Both species occupy a similar ecological niche but have been found to segregate spatially at a fine scale in the zone where they co‐occur (Aguilera et al., 2013). In this study, we test for the presence of hybrids among these two species in the contact zone. We also describe and compare the genetic structure of these sister species in an effort to begin understanding the evolutionary history that resulted in the current distribution of these species across a transition zone (Sousa & Hey, 2013).

## 2 METHODS

### 2.1 Sample collection and study

We collected muscle tissue samples from randomly selected *S*. *viridula* and *S*. *zebrina* specimens by hand from the mid‐upper intertidal zone of rocky shores during low tides. *S*. *viridula* samples were collected between Tocopilla (22°S) and Huentelauquén (31.6°S), and *S*. *zebrina* were collected between Huentelauquén and Chiloé (41.8°S) (Figure 1). Sampling site details as well as the number of samples per site are indicated in Table 1.

**TABLE 1:** Sampling information and genetic diversity summary statistics for each sampling location are estimated from species‐specific datasets

Sampling locality	Code	Lat, lon	*N* (put hyb)	*H_o_*	*H_e_*	*π*	*F*_IS_
***Scurria viridula***							
Tocopilla	TO	−22.11, −70.35	12	0.225^a^	0.218^a^	0.00043^a^	0.011^a^
Paposo	PA	−25.28, −70.45	12	0.230^ab^	0.224^ab^	0.00044^a^	0.014^a^
Temblador	TE	−29.47, −71.31	11	0.212^ac^	0.208^ac^	0.00041^ab^	0.020^a^
Talcaruca	TA	−30.49, −71.69	11 (5)	0.197^c^	0.189^c^	0.00039^b^	0.026^a^
Huentelauquén	HU	−31.63, −71.55	13 (1)	0.214^ac^	0.207^ac^	0.00040^ab^	0.012^a^
***Scurria zebrina***							
Huentelauquén	HU	−31.63, −71.55	12 (2)	0.219^a^	0.220^a^	0.00087^a^	0.035^a^
Puertecillo	PU	−34.05, −71.94	12	0.212^ab^	0.212^ab^	0.00084^a^	0.029^a^
Concepción	CN	−36.52, −72.95	12	0.208^b^	0.208^b^	0.00082^a^	0.031^a^
Loncoyén	LO	−39.81, −73.40	11	0.205^bc^	0.201^bc^	0.00080^a^	0.019^a^
Chiloé	CH	−41.84, −74.03	12	0.211^b^	0.209^b^	0.00082^a^	0.024^a^

Note

Different letters as superscripts indicate non‐overlapping 95% confidence intervals.

Abbreviations: (put hyb), number of putative hybrids according to the results of NewHybrids; *F*_IS_, inbreeding coefficient; He, expected heterozygosity; *H_o_*, observed heterozygosity; lat, latitude; lon, longitude; *N*, number of samples; *π*, genetic diversity.

### 2.2 Ethics statement

This research was undertaken in agreement with the policies and procedures of the Universidad Austral de Chile bioethical committee. Samples were collected under permit R. Ex N 2036, 2019 from the Subsecretaría de Pesca y Acuicultura (SUBPESCA) of the Chilean government.

### 2.3 RAD sequencing

DNA was extracted using the GenJet Genomic DNA purification kit (Thermo Scientific™, Waltham, MA, USA) following the manufacturer's protocol. Genomic DNA was shipped to Floragenex (Oregon, USA) where libraries were prepared using the restriction enzyme SphI, fragment size selection of 300–500 bp, and individual 10 bp barcodes for each sample. Two libraries were constructed and sequenced using Illumina 4000 technology, 101 bp single end.

Sequences were quality‐filtered and demultiplexed using the process_radtags module of STACKs vers. 2.53 (Rochette et al., 2019). Read length after trimming and filtering was 91 bp. Only reads with an average phred‐score >20 in a 10 bp window were kept for further analyses. Sequences with >1 bp ambiguity in barcodes or with one or more ambiguities in the restriction site were removed.

Demultiplexed reads were aligned to the reference genome of the congeneric species *Scurria scurra* (*unpublished data*) using bowtie2 (Langmead & Salzberg, 2012) with “‐sensitive” settings. Aligned read files were then used to generate a catalog of RAD loci using ref_map.pl in STACKs version 2.53. We then used the populations module in Stacks to calculate population genetic statistics and to filter loci. We kept only loci present in 95% of the samples, with a minor allele frequency of at least 3% and a maximum observed heterozygosity of 0.6. Only one SNP per locus was kept using the flag “—write‐random‐snp”. Individuals with an average coverage across loci below 5x were removed from the dataset. We produced three datasets with these settings: one including samples from both species, one including only *S*. *viridula* samples, and one including only *S*. *zebrina* samples. In both single species datasets, all putative hybrids were not included for analyses. Genetic summary statistics were estimated using the population module of STACKs.

### 2.4 Analysis of population structure

#### 2.4.1 Potential hybrids

Since these two species became in contact recently, we evaluated if there were genetic signals of hybridization. We expected that if hybrids exist, these should be found within the overlapping distribution area as *Scurria* limpets are considered to have low dispersal potential (Haye et al., 2014). To identify potential hybrids between species we analyzed the complete dataset (data from all individuals from both species) and performed a principal components analysis (PCA) using the function “glPca” from the Adegenet package (Jombart & Ahmed, 2011). We used LEA (Frichot & François, 2015) to estimate the ancestry of individuals under the hypothesis of two species (*K* = 2) with alpha = 10. We also used the program NewHybrids v1.1 (Anderson & Thompson, 2002) to estimate the posterior distribution that individual samples fall into different hybrid classes. Because NewHybrids cannot handle large numbers of loci, we filtered the full dataset and removed all loci with missing data, loci with minor allele frequency <0.4 (since the number of samples per species was similar, we expected a minor allele frequency ~0.5 for loci in which different alleles were fixed in each species). We also removed loci that were less than 500 kb apart. The filtered dataset used included 225 loci. Individuals of *S*. *viridula* from the northernmost sampling site (TO) and individuals of *S*. *zebrina* from the southernmost sampling site (CH) were assigned as pure individuals for each species, respectively. We used the R package ParallelNewhybrids (Wringe et al., 2017) to run NewHybrids in R. We ran the program five times using Jeffrey's prior for both allele frequencies (theta) and mixing proportions (pi) with a burn‐in of 20,000 followed by 50,000 sweeps.

#### 2.4.2 Population structure

We performed a PCA analysis for each of the two species separately as described in the previous section. We also ran LEA to estimate the most likely number of genetic clusters for each species by running the snmf algorithm for values of *K* = 1 to *K* = 10. For each species, we also tested for Isolation by Distance by running a Mantel test to estimate the correlation between a pairwise *F*_ST_ distance matrix and a Euclidean geographic distance matrix. Mantel tests were performed using the hierfstat R package with 1000 permutations for significance. We also performed an analysis of molecular variance (AMOVA) using the “poppr.amova” function from the poppr R package (Kamvar et al., 2014).

We tested for loci that exhibited significant deviations from neutral expectations in each species. We did this by performing two different outlier tests in each dataset. First, we used the R package “OutFLANK” (Whitlock & Lotterhos, 2015). After estimating *F*_ST_ values for all loci, we removed a fraction of loci from both the lower and upper ends of the *F*_ST_ distribution and trimmed loci with low heterozygosity. For *S*. *viridula* removing 5% of the lower and upper ends of the distribution and loci with heterozygosity <0.1 resulted in a good chi‐square distribution of *F*_ST_ values. For *S*. *zebrina*, removing 25% of the lowest distribution, 5% of the upper distribution, and loci with heterozygosity <0.1 reached a similar distribution of *F*_ST_ values. This reduced distribution was used to apply the likelihood function and infer the distribution of *F*_ST_ for neutral markers and determine outliers. The false discovery threshold was set to 0.05 to correct for multiple tests. Second, we used Bayescan 2.1 (Foll & Gaggiotti, 2008) to identify outliers. For this we ran the program setting the number of pilot runs to 5000, a burn‐in period of 50,000, thinning interval size was set to 10 and the prior odds for the neutral model was set to 100. We used a false discovery rate of 0.05.

## 3 RESULTS

### 3.1 Genetic diversity

Stacks assembled a total of 393,581 RAD‐tag derived loci. For the dataset including samples from both species, 8966 loci (for a total of 832,980 bp sequenced) passed population filter constraints (present in at least 95% of the samples, minor allele frequency >0.03, and maximum observed heterozygosity <0.6). Of these 5288 were variable. For the dataset including *S*. *viridula* samples only, 12,825 loci (for a total of 1,172,629 bp sequenced) passed population filter constraints. Of these 2188 were variable. For the dataset including *S*. *zebrina* samples only, 12,784 loci (for a total of 1,168,474 bp sequenced) passed population filter constraints. Of these 4424 were variable.

Summary statistics including observed (*H_o_*) and expected (*H_e_*) heterozygosity, *F*_IS_ and genetic diversity (*π*) per sampling location are presented in Table 1 and Appendix 1. Ho and He varied little among localities or species (min =0.197, max =0.230). Genetic diversity was similar among localities for both species (*S*. *viridula*: min =0.0004, max =0.00043, *S*. *zebrina*: min =0.0008, max =0.00087) but was slightly higher in the northern sampled localities in both species. Genetic diversity values in *S*. *zebrina* were on average two times higher than in *S*. *viridula*.

### 3.2 Potential hybrids

**FIGURE 2:**
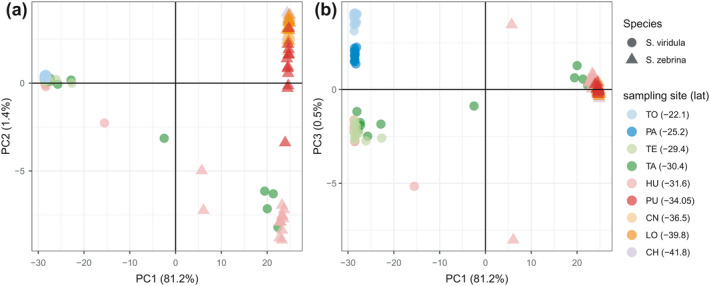
Principal components analysis (PCA) scatter plots. (a) the first (PC1) and second (PC2) components. (b) First (PC1) and third (PC3) (components) for the complete dataset. The percentage of variance explained by each PC is indicated in parentheses. Symbols represent individual genotypes of both species and colors indicate different sampling sites. Species names and sampling sites are indicated in the legend with the corresponding latitude indicated in parentheses

**FIGURE 3:**
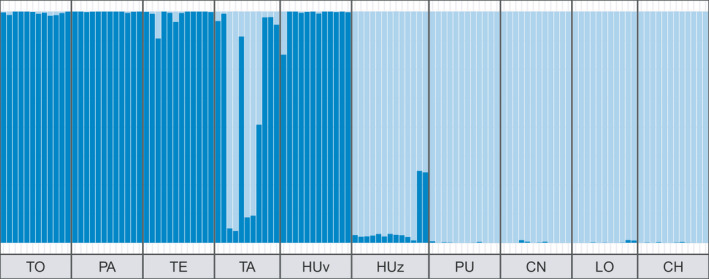
Barplot of individual ancestry proportions inferred with sNMF for *K* = 2. Codes in the bottom correspond to sampling sites in table one. Note that the “v” and “z” next to the code HU indicate *S*. *viridula* and *S*. *zebrina*, respectively

PCA results when considering the full dataset (both species) separated samples into three major groups. PC1 clearly discriminated between samples from both species, while PC2 separated two groups of populations within *S*. *zebrina* (Figure 2a). PC3 further discriminated against three groups of populations within *S*. *viridula* (Figure 2b). Interestingly, four individuals (two identified as *S*. *viridula* and two as *S*. *zebrina* from the TA and HU) fell in between the northern population group of *S*. *zebrina* samples and *S*. *viridula* samples group when plotting PC1 against PC2. Also, four samples classified as *S*. *viridula* in the field clustered with *S*. *zebrina* samples from the northern localities (HU and TA) (Figure 2a). LEA results for *K* = 2 were congruent with the PCA results (Figure 3). The results obtained with NewHybrids were congruent with the other approaches and all five runs produced similar results. Four samples were classified into a hybrid category: one *S*. *viridula* from TA as an F1 hybrid, one *S*. *viridula* from HU as a backcross (S. *viridula* × F1), and two *S*. *zebrina* samples from HU as backcrosses (*S. zebrina* × F1). All assignments had a posterior probability = 1.0.

### 3.3 Genetic structure

**FIGURE 4:**
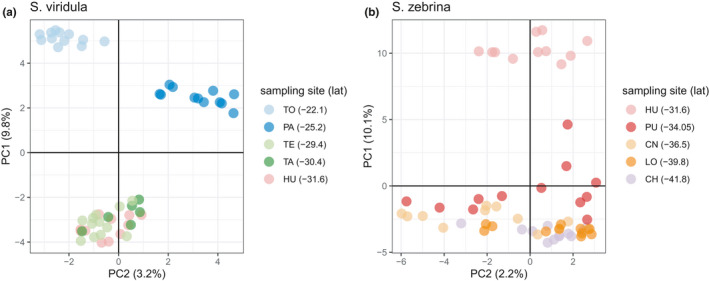
Principal components analysis (PCA) scatter plots. (a) *Scurria viridula*. (b) *Scurria zebrina*. The percentage of variance explained by each PC is indicated in parentheses. Symbols represent individual genotypes of both species and colors indicate different sampling sites. Species names and sampling sites are indicated in the legend with the corresponding latitude indicated in parentheses

For *S*. *viridula*, the results of the PCA separated individual genotypes into three clear groups (Figure 4a). One group consisted of only samples from the northernmost sampling site TO, the second group of samples composed of samples from PA, also in the north, and a third group composed of samples further south (TE, TA, and HU) (Figure 4a). For *S*. *zebrina*, the PCA revealed two groups of individual genotypes (Figure 4b). One group consisted of samples from HU, the northernmost sampling site. There was no clear grouping within the remaining samples.

**FIGURE 5:**
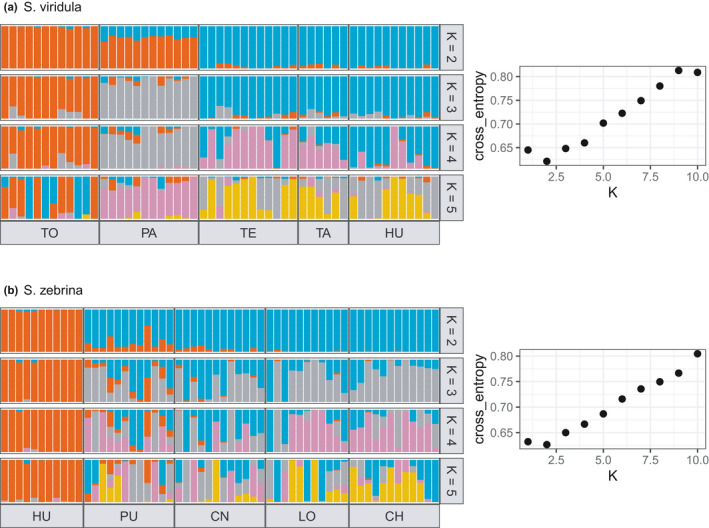
Barplot of individual ancestry proportions inferred with sNMF for *K* = 2–5 for each species. Panels on the left show the cross‐entropy values as a function of *K* (1–10)

Results from the sNMF genetic clustering suggested *K* = 2 as the most likely solution for *S*. *viridula* (Figure 5a, left panel) and were consistent with the PCA (Figure 5a, right panel). For *S*. *zebrina*, results from the sNMF genetic clustering suggested *K* = 2 as the most likely number of clusters (Figure 5b, left panel) and were consistent with the PCA (Figure 5b, right panel).

The AMOVA results showed that for *S*. *viridula*, 6% of genetic variation was explained by differences among populations (global *F*_ST_ = 0.060), 2% was explained by differences between samples within populations (*F*_IS_ = 0.020), and the rest of the variation was explained by variation within samples. For *S*. *zebrina*, results from AMOVA showed that 5.7% of genetic variation was explained by differences among populations (Global *F*_ST_ = 0.057) and 3.6% of the variation was explained by differences among samples within populations (*F*_IS_ = 0.037).

### 3.4 Isolation by distance

**FIGURE 6:**
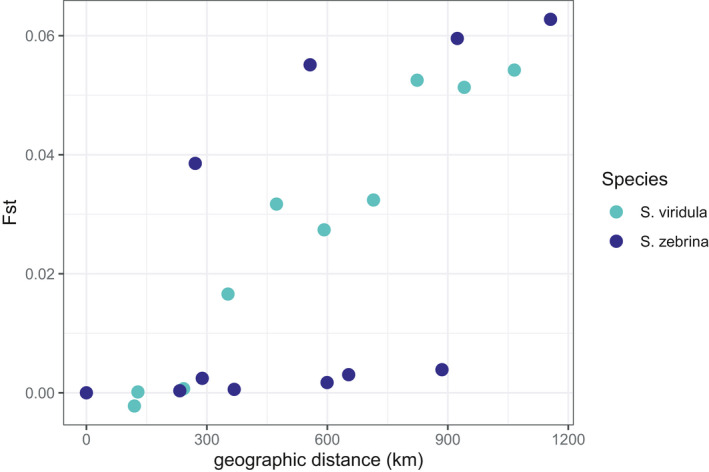
Correlation between genetic distance (*F*_ST_) and Euclidean geographic distance matrices for both species

There was a strong and significant correlation between the genetic distance and geographic distance matrices for *S*. *viridula* (Mantel *r* = 0.968, *p* = .0223). For *S*. *zebrina*, the correlation between genetic and geographic distance matrices was significant but weaker (Mantel *r* = 0.522, *p* = .008). A visual inspection of the correlation between genetic and geographic distance matrices clearly shows that for *S*. *viridula* genetic distance between populations increases linearly with geographic distance. For *S*. *zebrina*, this relationship is not linear (Figure 6).

### 3.5 Outliers

**FIGURE 7:**
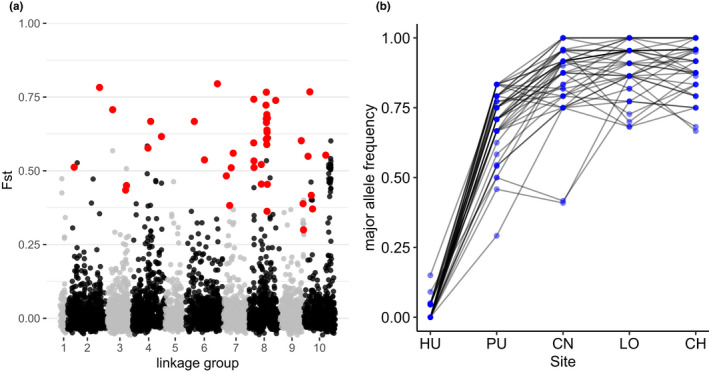
(a) Manhattan plot showing the individual SNP *F*_ST_ values for *S*. *zebrina* across the 10 principal linkage groups of the *Scurria scurra* genome. Points in red indicate putative outlier loci that were common to Outflank and Bayescan. (b) Allele frequency changes across the sampling sites ordered from north (HU) to south (CH). Blue dots indicate the estimated major allele frequency per population for each of the 43 outlier loci. Black lines describe allele frequency changes for each locus among sampling sites

The number of outliers varied substantially between species and methods. For *S*. *viridula*, no outlier loci were detected with Outflank and only three with Bayescan. In contrast, for *S*. *zebrina*, Outflank detected 143 outliers and Bayescan detected 43. All 43 outliers detected in Bayescan were part of the outliers detected in Outflank. These 43 outliers were distributed across the 10 main linkage groups of the *Scurria scurra* genome we used as a reference, but 11 of them clustered within a 1.8 Mb region within the linkage group 8 (Figure 7a). The major allele frequency distribution among sampling sites was very similar for all 43 outlier SNPs, having frequencies of less than 0.25 in the northernmost locality (HU), increasing to frequencies of 0.25–0.75 in PU, and frequencies higher than 0.7 in LO and CH (Figure 7b).

## 4 DISCUSSION

Our results indicate that two sister species with parapatric distributions, *S*. *viridula* and *S*. *zebrina*, appear to be hybridizing in the geographic zone where they overlap. Despite that both species display significant genetic structures within their geographic ranges, *S*. *zebrina* has twice the genetic diversity of *S*. *viridula*. Our results also indicate that the genetic structure of these two species is shaped by different evolutionary mechanisms. The genetic structure in *S*. *viridula* is consistent with a simple neutral genetic divergence model with isolation by distance, while *S*. *zebrina* appears strongly influenced by divergent natural selection (with several outlier loci identified by two different methods) with a genetic break at 32–34°S. Below, we discuss these results in the geographic context where these species are found. We also discuss the perspectives and novel opportunities this system offers to study hybridization and to better understand the role of evolutionary forces shaping genetic diversity in intertidal marine species (e.g. Ntuli et al., 2020; Zardi et al., 2007).

Our results strongly suggest that these two species hybridize in the geographic area where they co‐occur. While our sampling effort is not sufficient to characterize the degree and frequency of hybridization, our results do suggest that in this area, hybrid individuals with different degrees of introgression are rather common. Our results also indicate that introgressions seem to be occurring in both directions. These results suggest that neither of the species has developed efficient mechanisms of reproductive isolation. According to Rivadeneira and Fernández (2005) the endpoint of the distribution of *S*. *viridula* in 1962 was 29°55’, a latitude that does not overlap with the known *S*. *zebrina* distribution. This means that it is likely that these two species have been in contact only since *S*. *viridula* started expanding its range southward over the last decades. The fact that we found individuals with different degrees of introgression suggests that hybrids of these two species are fertile and reproductive isolation has not yet been achieved. Similar introgression patterns have been also reported for other congeneric marine gastropods in places where they occur in sympatry (Costa et al., 2020; Galindo et al., 2021; Hirase et al., 2021). *Scurria*, like most Patellogastropoda (Kolbin & Kulikova, 2011) are broadcast spawners and as such, the number and diversity of possible reproductive barriers might be limited compared with other organisms with different reproductive strategies (Nydam et al., 2017). Further studies that include laboratory crosses, genetic field surveys with higher sampling effort, and experiments to measure the performance of hybrids are needed to test these hypotheses.

The biogeographic zone between 30 and 35°S is characterized by a marked transition in oceanographic conditions, with a weak and persistent upwelling regime to the north and a variable strong upwelling to the south of this point and down to 39°S (Broitman et al., 2018; Hormazabal et al., 2004; Lara et al., 2019). This transition is also a biogeographic and phylogeographic break for numerous organisms including algae, intertidal barnacles, gastropods, and crustaceans (Ewers‐Saucedo et al., 2016; Haye et al., 2014, 2019; Sánchez et al., 2011; Thiel et al., 2007). Within both species, the major observed genetic partition clearly separated the region where both species overlap (30–34°S) from the rest of the samples. These genetic transitions coincide to some extent with two known biogeographic and phylogeographic breaks at 30 and 35°S, respectively (Lara et al., 2019). First, our results indicate that there is a significant genetic structure in *S*. *viridula* that separates Paposo (25°S) from the remaining sites to the south (29–31°S). This genetic transition coincides approximately with the 30°S biogeographic break. We note, however, that for *S*. *viridula*, the genetic composition of samples between 29 and 31°S is homogeneous and differentiation further north follows neutral expectations and isolation by distance. Our sampling design indicates that a genetic break in this species, if present, lies somewhere between 25 and 29°S. Yet, the clear pattern of isolation by distance and the lack of selection footprints suggests that genetic differentiation for *S*. *viridula* is rather gradual and could be explained simply by the limited dispersal potential in these organisms (Pinsky et al., 2017). Further sampling between 25 and 25°S would help elucidate if this is a genetic break or if genetic differentiation is indeed gradual. For *S*. *zebrina*, the genetic break occurs between 31 and 34°S and close to the biogeographic break around 35°S previously reported for benthic macroinvertebrates (Lancellotti & Vásquez, 1999) and that appears to be associated with major Andean river outflows (Lara et al., 2019). A phylogeographic break at 35°S has been reported in the ascidian *Pyura chilensis* (Quesada‐Calderón et al., 2021) and in the beach‐dwelling isopod *Excirolana hirsuticauda* (Haye et al., 2019). The latter species, *E*. *hirsuticauda*, has two phylogeographic breaks/transitions, one at ca. 30°S and the other at 35°S, coincident with the phylogeographic breaks for *S*. *viridula* and *S*. *zebrina*, respectively. It is interesting to note that the genetic break for *S*. *zebrina* around 35°S displays clear signals of natural selection. However, an alternative hypothesis (coupling hypothesis) is that intrinsic pre‐ or post‐zygotic genetic incompatibilities are in fact responsible for this genetic differentiation pattern but are coupled with exogenous barriers associated with ecological selection (Bierne et al., 2011). A more detailed sampling scheme around this genetic break would help to further explore this idea. Taken together, these results add to the growing body of the literature that shows that this coastal area around 30–35°S is a natural laboratory for the study of speciation in marine organisms.

Patterns of genetic diversity and genetic structure differed between species and suggest that these two species, despite their ecological similarities, differ considerably in the main evolutionary mechanisms that are shaping their current genetic structure. *S*. *zebrina* displayed consistently higher within population genetic diversity and significant signals of divergent natural selection in shaping genetic structure. In contrast, genetic diversity in *S*. *viridula* was half of what was observed in *S*. *zebrina*, and genetic structure followed neutral expectations. It is interesting to note that for both species within population genetic diversity diminishes gradually from north to south. Lower within population genetic diversity is often associated with the edge of the range of distribution (Eckert et al., 2008) and lower population sizes (Frankham, 1996; Willi et al., 2006). Thus, these results suggest that ancestral populations of both species originated at the northern end of their respective distributions and have expanded towards the south. Under this scenario, these species may have originated in allopatry and became in contact recently following the southwards expansion of *S*. *viridula* (Rivadeneira & Fernández, 2005). However, further analyses that test different demographic history alternatives will be required to test this hypothesis. Higher genetic diversity observed in *S*. *zebrina* suggests that this species might have larger effective population sizes. Previous studies have shown that both species have similar census sizes along most of their geographic distributions but that densities of *S*. *zebrina* increase threefold around 32–33°S (Aguilera et al., 2013, 2020). Interestingly, this area is adjacent to the genetic break we report for this species. This genetic break in *S*. *zebrina* (34–35°S) displays clear footprints of divergent selection, including 43 outlier loci. We also found that a large proportion of these outliers was concentrated in one of the linkage groups, a pattern that is consistent with the idea of divergent selection operating over a narrow genomic region (Nosil et al., 2009). In addition, we observed that for all outlier loci, one allele is fixed or near fixation at the locality north of the break compared to other localities further south. Previous studies have shown that the region between 30 and 33°S is characterized by strong between‐site differences in patterns of environmental variability associated with coastal upwelling circulation such as sea surface temperature, *p* co_2_ , and pH (Broitman et al., 2018; Lardies et al., 2021; Meneghesso et al., 2020; Navarrete et al., 2005). Taken together our results strongly suggest that the Huentelauquén population of *S zebrina* (31.6°S) could have adapted to the heterogeneous environmental conditions of this transition zone, which would explain its high densities, high genetic diversities, and natural selection footprints. We note, however, that it is also possible that these highly divergent regions could be shaped by differential introgression resulting from the hybridization of these two lineages and complex demographic histories (Duranton et al., 2018). Further studies with better genome coverage (i.e., whole‐genome re‐sequencing) will help distinguish these scenarios.

Previous studies that have evaluated phenotypic plasticity and responses of these two species to different environmental conditions from localities in this geographic zone where they overlap (30–33°S) have shown that *S*. *viridula* is more phenotypically plastic than *S*. *zebrina* in the region where both species reach similarly higher population densities when compared to the center of their ranges (Aguilera et al., 2020; Broitman et al., 2018; Lardies et al., 2021). In contrast, *S*. *zebrina* samples from Huentelauquén (31.6°S) were highly differentiated from other populations further south, away from this transition zone and this differentiation involves several loci that appear to be under divergent selection. Indeed, the phenotypic response of range edge populations at Talcaruca for *S*. *zebrina*, were significantly different from populations inside the range (Broitman et al., 2018). Our genetic results agree with the heterogeneous between‐site phenotypic responses reported previously for these species. Specifically, in studies of geographic variation in phenotypic plasticity of morphological and physiological traits of both species, which appear to correlate to the heterogeneous seascape of carbonate conditions and sea surface temperature across this biogeographic break in the SEP (Broitman et al., 2018; Lardies et al., 2021). Finally, further studies that evaluate phenotypic plasticity outside this transition zone for these two species and further genetic studies that reconstruct the demographic history of divergence of these lineages could help better understand the links between phenotypic responses and genetic structure.

## CONFLICT OF INTEREST

The authors declare no conflict of interests.

## AUTHOR CONTRIBUTIONS

**Pablo Saenz‐Agudelo:** Conceptualization (lead); Data curation (equal); Formal analysis (lead); Funding acquisition (lead); Investigation (equal); Methodology (equal); Project administration (lead); Resources (lead); Validation (equal); Visualization (equal); Writing – original draft (lead); Writing – review & editing (equal). **Livia Peluso:** Investigation (equal); Methodology (equal); Resources (equal); Writing – original draft (supporting); Writing – review & editing (equal). **Roberto Nespolo:** Conceptualization (equal); Formal analysis (equal); Investigation (equal); Writing – original draft (supporting); Writing – review & editing (equal). **Bernardo Broitman:** Conceptualization (equal); Data curation (equal); Investigation (equal); Methodology (equal); Writing – original draft (supporting); Writing – review & editing (equal). **Pilar A. Haye:** Investigation (equal); Resources (equal); Writing – original draft (supporting); Writing – review & editing (equal). **Marco Lardies:** Conceptualization (equal); Formal analysis (equal); Investigation (equal); Methodology (equal); Writing – original draft (supporting); Writing – review & editing (equal).

## Data Availability

Individual fastq data files are available at the SRA repository of NCBI under Bioproject number PRJNA825578. Raw SNP data for all three datasets used in this study is available at DRYAD (https://doi.org/10.5061/dryad.k6djh9w8p).

## APPENDIX 1

Plot of genetic diversity summary statistics. *H_o_*, observed heterozygosity; *H_e_*, expected heterozygosity; *F*_IS_, inbreeding coefficient; *π*, nucleotide diversity. In each plot mean values are indicated as a point and 95% confidence intervals around the mean are indicated as bars. The green color corresponds to *S*. *viridula* and the blue color corresponds to *S*. *zebrina*.
